# The Roles of Topoisomerases in Transcriptional Regulation

**DOI:** 10.3390/ijms27031552

**Published:** 2026-02-04

**Authors:** Kelli D. Fenelon, Ram Madabhushi

**Affiliations:** 1 Department of Psychiatry, Neuroscience and Cell Biology, University of Texas Southwestern Medical Center, Dallas, TX 75390, USA; kelli.fenelon@utsouthwestern.edu; 2 Peter O’Donnell Brain Institute, University of Texas Southwestern Medical Center, Dallas, TX 75390, USA

**Keywords:** topoisomerase, transcription, twin domain model, supercoiling, writhe, torsion, genome topology

## Abstract

Torsional stress from DNA supercoiling is receiving renewed attention as a driving force for chromosome folding and the establishment of gene activity states. Transcription is a major source of DNA supercoiling, while topoisomerases relax supercoils and solve topological problems that arise during DNA replication, transcription, and chromosome segregation. Recent technological advancements have allowed for the mapping of how torsional stress distributes within the genome and distinguishing between occupancy of topoisomerases on chromatin and sites where they are catalytically engaged. Coupling these innovations to assessments of 3D chromosome conformation and nascent transcription at high resolution have provided a new understanding of the relationships between supercoiling and topoisomerase activity. Here, we summarize the insights obtained from these recent studies and discuss how the interplay between transcription, supercoiling, and topoisomerases shapes cellular gene activity states.

## 1. Introduction

In its relaxed state, DNA predominantly adopts a right-handed double helical structure that is comprised of ~10.5 base pairs (bp) per helical turn. A consequence of the entwining of the two DNA strands is that cellular processes that require strand separation, such as DNA replication and transcription, dynamically generate torsional stress within the surrounding DNA [1,2,3,4].

Torsional stress can cause DNA to either become underwound or overwound compared to its normal helical pitch (twist) or cause DNA to buckle and loop onto itself (writhe). Mechanical energy introduced by supercoiling into DNA can affect the compaction and organization of chromosomes and the accessibility of DNA to transcription factors, while excessive supercoiling can also impede the movement of RNA and DNA polymerases [5,6]. A proper understanding of how cellular supercoiling levels are managed is therefore significant.

Whereas the activities of RNA and DNA polymerases, helicases, and ATP-dependent chromatin remodelers are major sources of DNA supercoiling in cells, topoisomerases are key enzymes that resolve torsional stress from supercoiling. Topoisomerases generate transient single or double strand breaks to open DNA gates through which they pass either a single-stranded or a duplex DNA segment, respectively. Following strand passage, topoisomerases reseal the DNA breaks they create and, in this way, solve topological problems that arise during replication, transcription, recombination, and chromosome segregation [2,3,6]. Mammalian cells express six distinct topoisomerases—TOP1, TOP2A, TOP2B, TOP3A, TOP3B, and TOP1mt. The structure–function relationships, biochemical activities, cellular functions of topoisomerases, and therapeutic applications of topoisomerase inhibitors in cancer have been discussed in many excellent reviews [5,6,7,8,9,10,11,12,13,14,15,16,17,18]. Here, we focus on the roles of topoisomerases in mammalian transcriptional regulation, drawing on insights gained from recent studies.

## 2. Relationships Between DNA Supercoiling and Transcription

**Figure 1:**
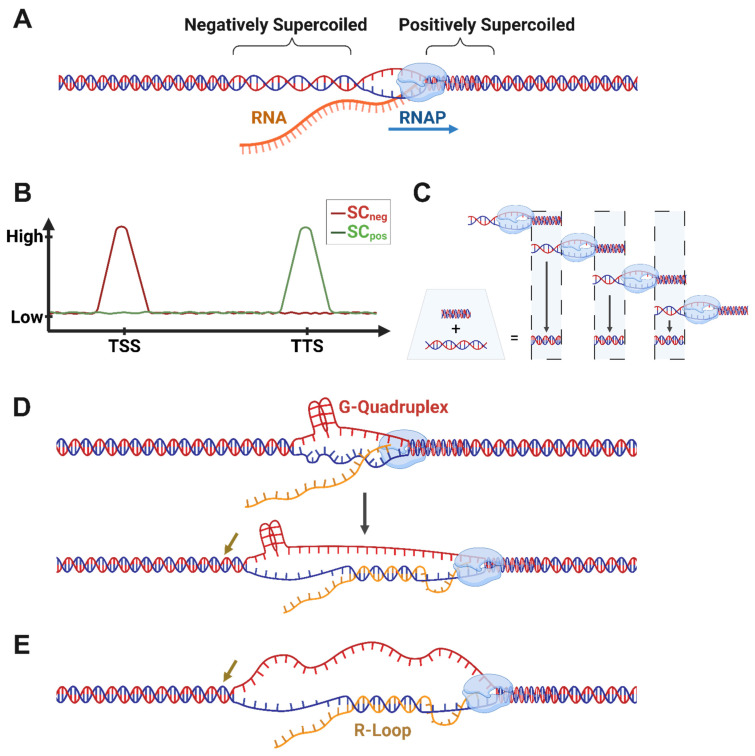
The twin domain model. (**A**) RNAP produces positive and negative supercoils ahead and behind it, respectively. This pooling of dynamic supercoiling may require topological restraints (e.g., TAD/subTAD boundaries). (**B**) The TDM predicts contrasting peaks of negative and positive supercoiling around the TSS and TTS, respectively, via cancelation effects of sequential RNAPs in the gene body (**C**). (**D**) In genes with G4-probable sequences near their TSS, negative supercoiling increases the likelihood of G4 formation, which leads to more thermodynamically permissive conditions for R-loop formation. (**E**) Excessive negative supercoiling creates conditions permissive to R-loop formation. Brown arrows indicate tempered underwinding resulting from secondary/tertiary DNA structure formation in the gene body. Created in BioRender. Fenelon, K. (2026) https://BioRender.com/mez0t4s (accessed on 29 January 2026).

The current understanding of supercoiling during transcription and the need for topoisomerases is predominantly based on the twin-domain model (Figure 1A–C). Proposed by Liu and Wang in 1987, the twin-domain model posits that RNAP movement during transcription causes DNA to become overwound (positively supercoiled) downstream of RNAP and underwound (negatively supercoiled) upstream [19]. The formation of supercoiling domains during transcription could potentially be prevented by the rotation of RNAP around the DNA; however, the model predicts that several factors, including the large size of RNAP, the RNA transcript, and factors bound to the nascent transcript could hinder RNAP rotation [19]. The twin-domain model and its implications for transcription-driven supercoiling have been supported by numerous observations [16,20,21,22,23,24,25], including the direct detection of supercoiling domains in a recent single-molecule study [26]. Surprisingly, although the RNA transcript significantly increased frictional drag in these experiments, RNAP was able to generate twin supercoiling domains even in the presence of RNase A, indicating that the resistance to rotation of RNAP could exist independently of the additional viscous drag caused by the nascent transcript [26]. An additional mechanism that could contribute to RNAP rotational inertia in a subset of genes is the recent finding that DNA topology near the promoters of genes can act through a sequence-autonomous feedback mechanism to regulate transcription, whereby the negative supercoiling at the promoter, which reduces the DNA melting temperature thereby promoting RNAP recruitment, is attenuated by the formation of negative-supercoiling-facilitated G-quadruplex formation on the non-template strand in a sequence dependent manner which instigates R-loop formation on the template strand, impeding RNAP recruitment and suppressing transcription (Figure 1D) [27]. While the mechanisms that prevent RNAP rotation are still incompletely understood, these studies establish that transcription is a major source of dynamic supercoiling.

**Figure 2:**
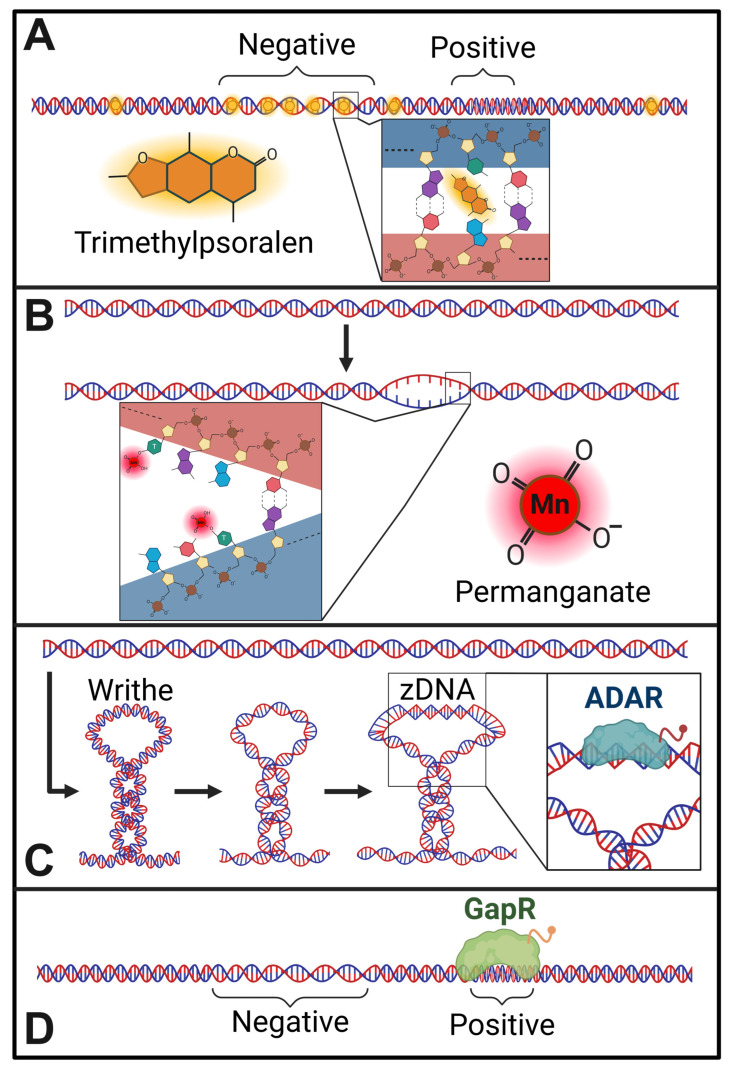
Supercoil labeling. Being able to identify supercoiled regions of the genome *in vivo* is paramount to understanding the roles supercoiling and topoisomerases play in genomic regulation. However, doing so in efficient, reliable ways has been challenging, with most effective methods having been developed in the most recent couple of decades. Modern techniques allow for locating these topological structures by high-throughput sequencing following structural labeling. Negative supercoiling can be labeled (**A**) by intercalating trimethylpsoralen (TMP), which preferentially intercalates into negatively supercoiled DNA, into the genomic DNA of treated living cells followed by crosslinking of the TMP to the DNA with ultraviolet radiation [28,29,34]. Single-stranded DNA, which is a feature of complex DNA superstructures and can result *de novo* from negative-supercoiling-induced melting, can be labeled (**B**) by treating and crosslinking the genome with potassium permanganate [36,37,38]. In regions of extreme negative supercoiling, writhe can be insufficient to absorb the resultant torsion and zDNA, left-handed DNA helicity, can form, which is labelable (**C**) using the Zaa domains of ADAR proteins [39,40]. On the other hand, methods for labeling positive supercoiling have proven more challenging, but recent advancements show significant promise (**D**) using the yeast GapR protein, which preferentially binds positively supercoiled, double-stranded DNA [20,41,42]. Created in BioRender. Fenelon, K. (2026) https://BioRender.com/wb3t3e6 (accessed on: 29 January 2026).

An immediately recognized implication of the twin-domain model is that transcription could determine the steady-state level of supercoiling within cells [19]. Additionally, Liu and Wang also postulated that transcription-generated supercoiling could act as a driving force for structural transitions in the genome [19]. Both predictions have been validated through subsequent studies. For instance, the intercalation of psoralen analogues has been widely used to map the distribution patterns of underwound (negatively supercoiled) DNA in yeast, insect, worm, and mammalian cells (See Figure 2) [20,21,24,28,29,30,31,32,33]. The psoralen derivative, 4,5′,8-trimethylpsoralen (TMP), is a cell-permeable molecule that preferentially intercalates within underwound DNA and forms interstrand crosslinks when cells are exposed to 365 nm UV light [29,34]. Various strategies have been employed to isolate and sequence psoralen-incorporated DNA and assess the distribution of negatively supercoiled DNA under distinct cellular conditions. Importantly, in such experiments, inhibition of transcription caused a significant reduction in TMP incorporation, indicating that transcription is a major contributor to steady-state supercoiling levels [21]. Similarly, supercoiling was shown to cause the formation of non-B form DNA at specific loci (See Figure 2) [22,35]. Several other effects of supercoiling on chromosome organization are considered below.

Whereas transcription is a major source of supercoiling, many aspects of transcription are also affected by supercoiling. For instance, negatively supercoiled DNA has a configuration that facilitates the binding of transcription factors and the assembly of nucleosomes, whereas positively supercoiled DNA formed ahead of RNAP can cause nucleosome eviction to facilitate RNAP movement [4,43]. High levels of supercoiling can also cause the stalling of RNAP [44]. Supercoiling could also influence gene transcription by modulating interactions between genes and distal gene regulatory elements and environments, such as enhancers, silencers, or the nuclear lamina [43,45]. Overall, the effects of supercoiling depend on several factors, including the rate at which they are generated (by RNAP or other factors), the extent to which supercoils either propagate or are constrained, and the activities of topoisomerases, which resolve supercoils. Some observations suggest that transcription-generated supercoiling is largely a local phenomenon and a short-range force [20,21]. For instance, recent analysis of TMP-crosslinked regions by next-generation sequencing in yeast revealed that differences in torsion were largely restricted to regions between 1 and 2 kb surrounding transcription units [20]. Such distribution patterns are sufficient to explain observations in which transcription-generated supercoiling in one gene affects the transcription of neighboring genes [20]. Consistent with the idea that negative supercoiling upstream of transcription start sites favors transcription initiation, increased supercoiling elevated transcription of divergent gene pairs in *C. elegans* [46]. Short-range distribution of supercoiling and formation of writhe has also been proposed to explain how some yeast genes are topologically insulated by stable negatively supercoiled regions, which prevent diffusion of torsional stresses and produce gene-locus-solitary topological domains [20]. The phenomenon of coupled transcription linked to shared topology between nearby genes is a topic of interest in prokaryotic research also, where transcription has been shown to be a short range force transmitter [47,48]. This sharing of supercoiling is likely to have gene regulatory effects paralogous to the inducement of transcriptional bursting in bacterial operons [49,50].

In contrast to transcription-generated supercoiling being a largely short-range force, other reports suggest that transcription-generated supercoiling distributes over megabase-scale distances to form large supercoiling domains that correspond roughly to chromosome compartments [24,31,33]. Transcription-generated supercoiling was also found to propagate tens of kilobases in bacteria. One reason for these differences is that different psoralen analogues (TMP, biotinylated TMP, ATMP), sequencing (array-based methods vs. next-generation sequencing), and data normalization strategies (to account for sequence and accessibility bias of TMP incorporation) were employed to interpret the distribution of TMP signals. Alternatively, distinct properties of chromosomes, such as the density of transcription units and topological domain organization could distinctly affect the distribution of transcription-generated supercoiling. The continued generation of high-resolution maps of supercoiling distribution across cell types and tissues, as well as the development of new methods to assess the distribution of positive supercoiling and writhe should clarify this issue (See Figure 2).

## 3. Topoisomerases and Their Impact on Cellular Functions

**Figure 3:**
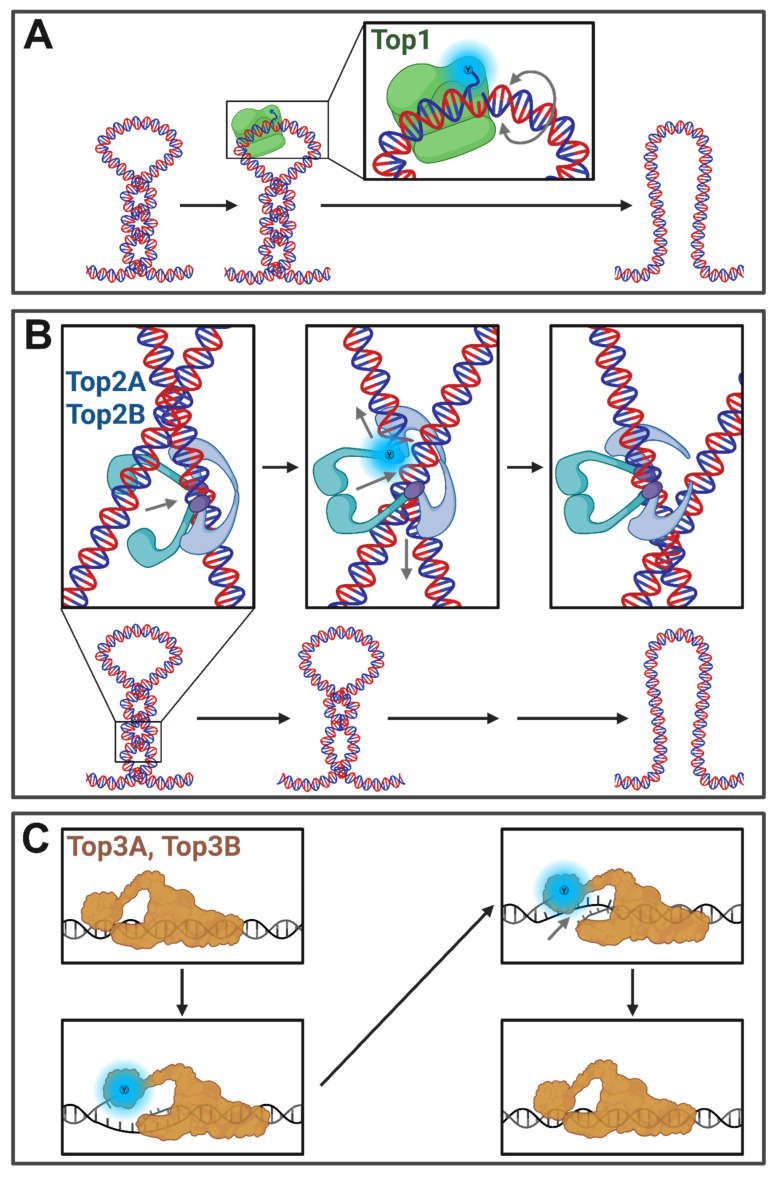
Mammalian topoisomerase mechanisms of action. (**A**) TOP1 severs one strand of the DNA helix and allows for supercoil relaxation through free rotation of the strand before religating. (**B**) TOP2 enzymes induce a DSB and pass a second intact double strand through the gap before religating the DSB. (**C**) TOP3 enzymes create a single-stranded break of either DNA or RNA and pass the complementary strand through the break before relegation. Radiating blue circles denote tyrosine residues used to temporarily facilitate covalent bonding to the severed ends of the DNA/RNA. Grey arrows denote strand dynamics. Created in BioRender. Fenelon, K. (2026) https://BioRender.com/l3b7lm7 (accessed on 29 January 2026).

Mammalian cells carry six topoisomerase genes: *TOP1*, *TOP1(mt)*, *TOP2A*, *TOP2B*, *TOP3A*, and *TOP3B* as well as the topoisomerase-like *SPO11* (See Table 1). TOP1, a type IB topoisomerase conserved across eukaryotes, is a ubiquitously expressed “housekeeping gene”, which resolves both positive and negative supercoils by nicking one strand and allowing rotation to resolve torsion before religating the nick (Figure 3A) [51,52]. TOP2A and TOP2B evolved from Top2 of lower eukaryotes [53]. Both TOP2A and TOP2B are type IIA topoisomerases that resolve catenated DNA and supercoiled DNA by making a double-stranded break and passaging a second strand through the break before rejoining and ligating the severed strand (Figure 3B). TOP3A and TOP3B are type IA topoisomerases, which have received far less attention in their function than TOP1, TOP2A, and TOP2B [54]. TOP3A is known to function with BLM helicase to resolve DNA recombination intermediates [55] and to resolve DNA structural aberrances within the mitochondria [56] while TOP3B is implicated in transcriptional regulation and R-loop resolution (Figure 3C) [57,58,59,60,61,62,63]. TOP1MT is a type 1B topoisomerase expressed in mitochondria [64]. SPO11 is a topoisomerase-like enzyme, similar to type IIB topoisomerases, which creates double stranded breaks to facilitate recombination in meiotic cells [65].

Ubiquitously expressed TOP1 is the primary enzyme responsible for resolving transient supercoils generated both by transcription and replication [6,76,77]. Because TOP1 is most efficient on naked nucleosome-free DNA, its primary role tends to be in resolving negative supercoiling, trailing actively transcribing RNAP [78,79]. This function is crucial for preventing R-loops but comes with the hazard of collision with subsequent polymerases, which can lead to toxic DSBs (Figure 1E) [57,80,81,82]. TOP1 is essential for development and cell survival [66,83,84,85]. Furthermore, the targeted depletion of TOP1 through cKO or inhibition in neurons causes neurodegeneration, in multiple cancer cell lines, it reduces cancer cell fitness, and in immune cells, it causes impaired recombination and reduced survival [86,87,88,89,90,91].

The Gnathostomata Top2 duplication likely allowed TOP2A to specialize in cell division [92]. TOP2A expression and function are closely coupled to cell proliferation, with low expression in quiescent cells, rising levels in S phase, and peak expression in G2 and M phases [93,94]. TOP2A’s primary and essential roles are to relieve topological stresses generated by DNA replication and to decatenate sister chromatids. Yeast TOP2A paralog, Top2, also functions with HMGB protein, Hmo1, to ensure chromosome integrity at sites of S-phase transcription, and human HMGB1/2 expression correlates with and induces TOP2A expression [95,96]. Inhibiting the catalytic activity of mouse TOP2A and TOP2B with ICRF-193 induces DNA damage in the genome in a cell-cycle dependent manner predominantly through TOP2A function [97], likely as part of the mitotic handoff from TOP2A to TOP2B is necessary to resolve residual condensin-dependent topological entanglements [98].

TOP2B is expressed more broadly than TOP2A and is found in both proliferating cells and post-mitotic cells [93,99]. Knockout mouse models show TOP2B is essential for certain gene regulatory regimes, including for neuronal development, e.g., axon guidance, and is required for proper B-cell development in the immune system [93,100]. TOP2B’s essential developmental role is most prominent in the nervous system, where it is required for the expression of developmentally regulated genes [101] and is highly expressed in Purkinje cells, the cerebellar granule, and other differentiating cells [102]. Further, it is crucial for neuronal survival [103] and the lamina-specific targeting of retinal ganglion cell axons [104]. In humans, *de novo* mutations in TOP2B have been linked to developmental disruption and intellectual pathology [105]. Interestingly, KD of TOP2 by CRISPR in juvenile mice extended their lifespan and improved their health, a phenomenon the authors replicated in Top2 KD yeast and *C. elegans* [106]. TOP2B tends to act at loop anchors co-occupied by CTCF and cohesin [107] and is implicated in creating persistent DSBs and potentially even clipping genomic loops [107]. Indeed, TOP2B has been shown to physically interact with cohesin and CTCF and often colocalizes with them at TAD boundaries [108,109]. TOP2B’s C-terminal domain (CTD) facilitates its proper function via degradation of its closed-clamp intermediate (TOP2Bcc) [110]. Kawano and Ikeda showed that closed clamp degradation is prevented by truncating the C-terminus of TOP2B and induced in TOP2A by replacing the TOP2A CTD with the TOP2B CTD. This finding helps support the proposition that the divergence in CTD sequence is the primary determinant of the isoform-specific functions of TOP2A and TOP2B [111]. This is further supported by the TOP2A-CTD-specific catalytic activity association with RNA [112] and disordered-domain-dependent substrate selection [113]. In contrast, TOP2B’s CTD has been shown to act as a regulatory domain for both enzymatic activity [114] and DNA binding [115].

While TOP3A is essential, being vital to proper mitochondrial function and telomere maintenance, TOP3B is largely dispensable [73,74,116,117,118]. *Top3b* KO mice survive, but they have shorter lifespans, increased autoimmunity, reduced synaptogenesis, age-coincident lesions in several internal organs, reduced fertility, aneuploidy, chronic inflammation, and immune dysregulation, while cell and disease studies report defective activation of p53 DNA damage response, accelerated cancer progression, and genomic instability [62,75,119,120,121,122,123,124].

## 4. Supercoiling Resolution by Topoisomerases and Their Roles in RNAP Movement

Topoisomerases are extremely effective at resolving supercoiling *in vitro* and are abundantly expressed in cells. Based on this and observations that a buildup of supercoiling can stall RNAP *in vitro*, it is thought that a crucial function of topoisomerases in transcription is to prevent supercoil buildup to levels that can stall RNAP [44]. However, it is unclear from recent studies whether supercoil accumulation even at highly transcribed genes in the absence of topoisomerases is sufficient to stall RNAP, especially in long mammalian chromosomes. For instance, gene expression analyses in cultured cortical neurons indicated that the cellular topoisomerases, TOP1 and TOP2B, were dispensable for the transcription of most genes but were important for the transcription of a small set of very long genes [84]. Similar results were reported for proliferating cells [125]. In fact, overexpression of wild-type TOP1 caused a significant downregulation of more than 7000 genes in HEK293T cells [126]. Knocking-in a mutant TOP1 that displays reduced chromatin binding was correlated with increased levels of transcriptionally engaged RNAPII, as determined by PRO-seq and levels of elongating RNAPII by ChIP-seq [126]. This is despite observations that even transient TOP1 inhibition results in increased negative supercoiling genome-wide [21,24,33]. Likewise, inhibition of TOP2, which results in smaller increases in negative supercoiling also causes either a downregulation in steady-state transcripts of only a few genes or both upregulation and downregulation of genes [21,24,33,103,127].

Although the assessment of steady-state transcript levels is useful, it does not address the issue of whether transcriptional changes observed following topoisomerase inhibition and knockdown result directly from effects on transcriptionally engaged RNAPs. The utilization of nuclear run-on (GRO/PRO-seq and related assays) experiments has begun to provide insights into this issue. For instance, global run-on sequencing in cultured mouse cortical neurons following TOP2B inhibition for 30 min revealed that the levels of transcriptionally engaged RNAPII were generally elevated and not diminished at transcriptional units [128]. Similar results were reported from nascent RNA-seq experiments in fly S2 cells following TOP1 inhibition [24]. Taken together, the results of nascent and steady-state RNA-seq experiments suggest that transcription-generated torsional stress in the absence of topoisomerases is not generally sufficient to stall RNAP movement at most genes and could in fact support transcription, at least in the short run. Such a scenario is consistent with several transcription-supporting functions of increased supercoiling, such as the displacement of nucleosomes by positive supercoiling ahead of RNAP and the melting of promoter DNA by negative supercoiling behind RNAP. They are also consistent with the interpretation from TMP incorporation studies that supercoils propagate over large distances from sites of transcription. Propagation of positive supercoiling could be accommodated by chromatin ahead of RNAP, while negative supercoiling could also propagate either linearly or by “hopping” over large distances [24,25].

**Figure 4:**
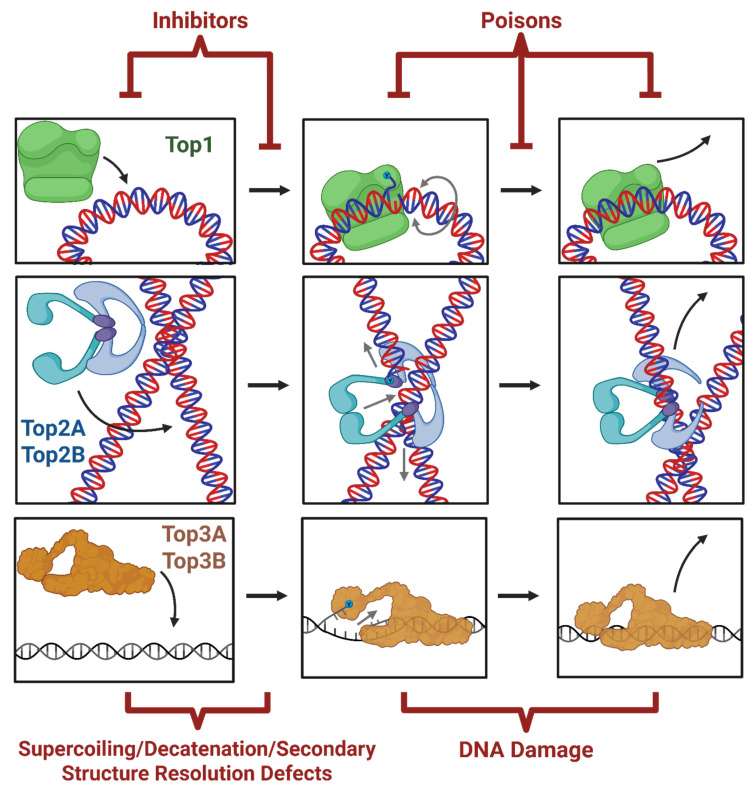
Topoisomerase inhibition. a wide variety of compounds, drugs, and genetic tools (see Table 2) have been developed to inhibit (prevent) or poison (disrupt) topoisomerase function [158]. Created in BioRender. Fenelon, K. (2026) https://BioRender.com/gwm6a0m (accessed on 27 January 2026).

The benefits of torsional stress for transcription raise the issue of whether topoisomerases are regulated to allow torsional stress to manifest its effects. Early recombination-based studies suggested that topoisomerase activity is unable to keep up with transcription-generated supercoiling [22,35]. Similarly, TMP incorporation is markedly reduced upon transcription inhibition, indicating that topoisomerases only resolve a fraction of transcription-generated supercoiling. Yet whether this deficit in supercoil resolution occurs despite “maximal” topoisomerase activity or whether available topoisomerase activity is somehow curtailed remained unclear. Analysis of topoisomerase occupancy patterns on chromatin (using ChIP-seq) and comparison with patterns of catalytically engaged topoisomerases (TOP1 CAD-seq, TOP2cc-seq, END-seq, etc.) have provided new insights into this problem [107,128,129,130]. As mentioned above, topoisomerases generate transient DNA breaks as intermediates in their catalytic cycle, in which they become covalently attached to DNA ends. The formation of such covalent cleavage complexes (TOP1ccs and TOP2ccs) can thus be used as a readout of their catalytic engagement. Exploiting this principle, cells are treated with topoisomerase poisons, such as camptothecin (to trap TOP1) and etoposide (to trap TOP2) (See Figure 4 and Table 2 for a comprehensive list of topo inhibitors and poisons and their mechanisms). TOP1ccs and TOP2ccs are then isolated, processed, and sequenced using various strategies to determine their distribution genome-wide. Using such methods, studies in HCT116 human colorectal carcinoma cells revealed that, although the occupancy of TOP1 is enriched at transcription start sites of actively transcribed genes, it is largely held in a catalytically inactive state [129]. Similar results were observed for TOP2B in cultured mouse cortical neurons [128]. The relative depletion of topoisomerase activity at transcription start sites could explain how negative supercoiling could persist in these regions and allow for both the binding of transcription factors and the pausing of RNAP in promoter-proximal regions. By contrast, levels of catalytically engaged TOP1 and TOP2 were found to be elevated within gene bodies and tracked with the level of transcription [128,129,130]. Intriguingly, analysis in neurons suggested that although levels of TOP2Bccs within gene bodies correlate with their level of transcription, this relationship was lost in gene bodies that lacked chromatin markers usually found in the bodies of actively transcribed genes, such as H3K36me3 [128]. These results suggest that posttranslational histone modifications could affect the catalytic engagement of TOP2B within gene bodies and underlie their attunement to the level of transcription. TOP2B activity was shown to be rapidly modulated in response to a variety of external stimuli and facilitate the transcription of stimulus-responsive genes, as described below. Overall, these results indicate that topoisomerases are utilized more selectively than previously thought, which, in turn, could allow for the torsion-dependent regulation of transcription through previously underappreciated mechanisms.

In this regard, recent results suggest a need to revisit the observed requirement of topoisomerases for supporting the transcription of long genes (>80–100 kb) [84,125]. Based on the twin-domain model, it is widely interpreted that the transcription of long genes could cause significant accumulation of supercoiling that could ultimately stall RNAP progression in the absence of topoisomerases. However, TMP incorporation signals from several studies are inconsistent with long genes accumulating significant levels of supercoiling in their gene bodies compared to other short or highly transcribed genes, whose transcription is independent of topoisomerases [20,33]. Instead, a recent study suggests that the dependence of long gene transcription in neurons on topoisomerase activity could be related to the presence of intragenic enhancers [128]. Analysis of chromatin states within the gene bodies of neurons revealed an enrichment of enhancer like states within the bodies of genes longer than 80 kb. To understand how the presence of intragenic enhancers affects long gene transcription, GRO-seq signals were examined in regions proximal and distal to the sites of intragenic enhancers following TOP2B inhibition. Interestingly, GRO-seq signals were unaffected by the presence of intragenic enhancers in untreated neurons. By contrast, GRO-seq signals were reduced in regions distal to intragenic enhancers compared to regions proximal to intragenic enhancers when TOP2B was inhibited. Moreover, when long genes were filtered based on the presence of intragenic enhancers, long genes that contained intragenic enhancers were downregulated following TOP2B inhibition compared to long genes that lacked intragenic enhancers [128]. Separately, analysis of GRO-seq signals revealed that inhibiting TOP2B stimulated transcription initiation not only at transcription start sites of most genes but also across enhancers and other cryptic sites, potentially from increased supercoiling [128]. Transcription from intragenic sites interferes with transcription of their host genes, likely through collisions between RNAPs [151]. The enrichment of intragenic enhancers and other cryptic transcription sites within long genes could explain why these genes are reliant on topoisomerases, which could suppress cryptic transcription initiation by controlling genome-wide supercoiling levels. As mentioned above, levels of TOP2Bccs were elevated in H3K36me3-rich chromatin. A major function of H3K36me3 is to suppress cryptic transcription, especially in long and infrequently transcribed genes [152,153,154,155,156,157]. Overall, these results suggest a model in which TOP2B recruited to H3K36me3-rich chromatin suppresses cryptic transcription within long neuronal genes by resolving supercoiling. It will be interesting to test whether TOP1 has roles in suppressing cryptic transcription and whether similar mechanisms could explain the reliance of long neuronal genes on TOP1 for their transcription.

## 5. Topoisomerase Regulation of Gene Activity Patterns Through Genome Organization

While the molecular mechanisms of gene regulation have been investigated extensively, several developments indicate how the three-dimensional (3D) organization of the genome governs gene activity. Advanced imaging approaches have discovered that spatial organization of the genome in eukaryotic cells is nonrandom and that chromosomes and genes are radially arranged in statistically preferred positions within the nucleus [159]. The preferred radial positioning of genes can vary according to the cellular type and activity states, and relocating genes to distinct genome neighborhoods can affect their transcription [159]. These observations suggest that mechanisms that control gene positioning within the nucleus could exert a regulatory effect on gene activity. Additionally, spatiotemporal control of gene expression patterns is largely dependent on the activities of enhancers [160]. However, because enhancers are often located at large genomic distances from their target genes, their precise mechanism of action was initially unclear. The use of 3C-based assays, which identified long-range interactions between distally located enhancers and promoters, clarified this issue and demonstrated that chromatin looping plays a major role in gene regulation through enhancer–promoter coupling [161]. 3C-based experiments, such as Hi-C and Micro-C, have also generated high-resolution maps of chromatin contacts genome-wide. These studies revealed that chromosomes are folded into units of frequently self-interacting chromatin segments called topologically associated domains (TADs) that range from ~200 kb to 1Mb in size, and transcriptionally active and inactive regions organize into distinct chromosome compartments [162]. Whereas TADs themselves tend to be invariant across cell types, loop domains of enriched and dynamic contact frequencies are observed within TADs. It is thought that TADs govern gene activity by functionally compartmentalizing chromatin contacts and either promoting or constraining interactions between gene regulatory regions and genes [162]. Together, these studies emphasize the importance of 3D chromatin organization for gene activity and suggest that mechanisms that affect chromatin interactions could significantly affect the elaboration of gene activity states. Because supercoiling introduces free energy into DNA, it is appealing to consider that this energy could affect chromosome dynamics and gene activity patterns.

As mentioned above, observations that TOP2 interacts with CTCF and cohesin and that cohesin affects the recruitment of TOP2 have led to the idea that TOP2-mediated resolution of torsional stress could affect chromosome organization into loops and TADs [5]. This idea was tested in a recent study in which chromosome contacts were assessed following the depletion of TOP2A and TOP2B in human HCT116 cells using Hi-C and Micro-C [163]. Furthermore, the distribution of positive supercoiling was assessed using GapRUN. These studies revealed that positive supercoiling is also distributed at gene boundaries and around anchor sites of chromosome loops [163]. TOP2 isoforms were also enriched at these regions. Interestingly, depletion of both TOP2A and TOP2B simultaneously led to increased higher-order chromosome interactions within megabase scale regions [163]. Regions with affected interactions were positively supercoiled and enriched for the boundaries of nuclear lamina-associated domains (LADs) and highly transcribed regions. Overall, these results suggest that TOP2 could have a role in regulating interactions between LADs and non-LADs [163]. How positive supercoiling accumulates at LAD boundaries is not unclear but would be consistent with a model in which supercoils propagate from sites of high transcription to distal regions. These results are also consistent with results from other studies, which indicate that TOP2B interacts with nuclear scaffold/matrix proteins [164,165]. In human retinal cells, it was recently shown that TOP2B is required for proper LAD formation and that supercoils pool in iLADs [166]. Interestingly, Lamin B receptor (LBR) knockout produces a similar LAD landscape to that of TOP2B depletion. LBR is a key member of the nuclear lamina that interacts with heterochromatin [167] and links epigenetically marked chromatin to the nuclear periphery [168]. Furthermore, it is known that LBR binding is topology sensitive, preferentially binding non-BDNA linker DNA [169].

**Figure 5:**
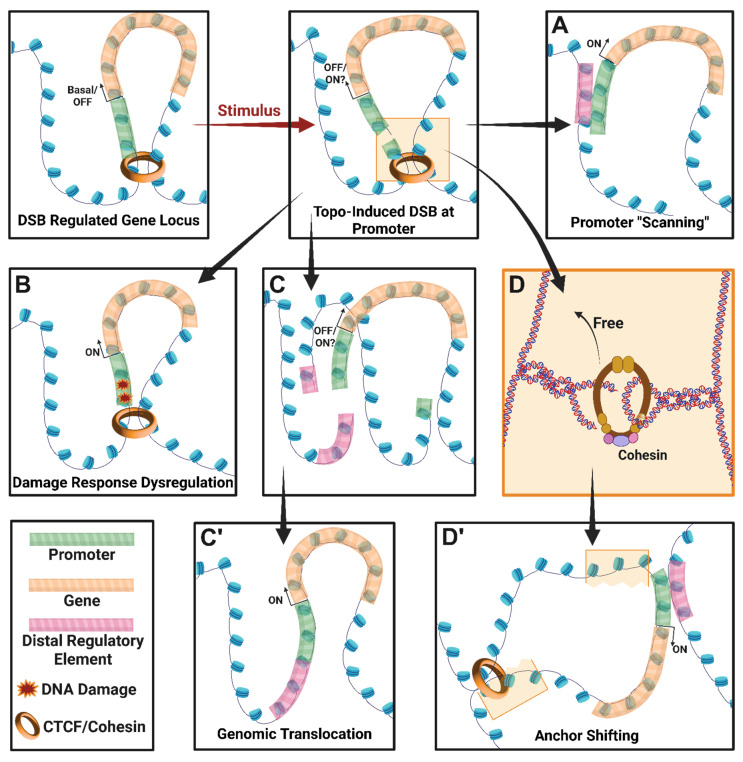
DSB transcriptional control models. When a DSB associated with transcriptional control occurs near a promoter, transcription may be affected in several ways. (**A**) DSBs may allow for severed ends to “scan” or freely float to find “sticky” E-P contacts. (**B**) DNA damage response may induce altered gene expression. (**C**,**C**’) A translocation may introduce a regulatory element in proximity to the promoter. (**D**,**D**’) The DSB may release trapped cohesin complexes facilitating E-P contacts otherwise spatially restrained. Created in BioRender. Fenelon, K. (2026) https://BioRender.com/0smajic (accessed on 30 January 2026).

In addition to remodeling 3D chromosome architecture by resolving supercoiling, TOP2B could also affect such contacts through the formation of stimulus-induced DNA double strand breaks (DSBs). First described in response to estrogen stimulation, studies performed across many cell types have described that TOP2B generates stimulus-induced DNA DSBs within the promoters of specific stimulus-responsive genes [109,170,171,172,173,174,175,176,177]. For instance, in neurons these stimulus-induced DSBs are enriched within promoters of neuronal early response genes (ERGs), such as *Fos*, *Npas4*, *Egr1*, *Nr4a1*, and *Arc*, whose products mediate experience-dependent synaptic changes and the development of lasting adaptive behaviors, such as learning and long-term memory formation [109,178,179,180]. The formation of such stimulus-induced DSBs facilitates the rapid transcription of neuronal ERGs, yet how DSB formation stimulates ERG transcription is not fully understood (Figure 5). A recent preprint provides some clues to this issue [181]. Neuronal ERGs are already primed for rapid transcription even under basal conditions but require the formation of enhancer–promoter contacts following stimulation. Chromosome conformation capture experiments revealed that DSB formation within ERG promoters alone is sufficient to mimic the contact patterns observed following neuronal stimulation [181]. Furthermore, recurrent cycles of DSB formation and repair progressively remodeled chromosome contacts at ERG promoters and potentiated ERG transcription in response to ensuing rounds neuronal stimulation [181]. These effects, which resembled the formation of transcriptional memory, indicate how TOP2B-mediated DSB formation could shape gene activity patterns and positions TOP2B as a potential regulator of the nexus between 3D genomic architecture, mechanical tethering to the nuclear lamina, and gene expression [182,183,184,185].

## 6. Conclusions

Recent studies indicate a pivotal role for torsional stress from DNA supercoiling in shaping gene activity states [174,186,187], by either directly modulating the binding of transcription factors [83], transcription initiation, and RNAPII movement or indirectly, by altering chromosome architecture [45,188], which in turn, affects the interactions of gene regulatory elements with cognate genes. Topoisomerases resolve torsional stress from supercoiling but seem to be utilized in ways that allow for the effects of torsional stress to dynamically manifest within the genome. In the future, continued innovations in the ability to measure the distribution of positive supercoils, writhe, and non-B DNA structures, as well as a betterer understanding of mechanisms that regulate topoisomerase activities should clarify how torsional stress manifests within the genome and regulates genome function.

While this review focuses on mammalian topoisomerases, the evolutionary history, expression patterns, and roles of topoisomerases across the kingdoms of life are complex [11]. Mammalian TOP1 likely evolved from the ancestral Type IB enzymes conserved from yeast and some viruses [189,190]. Mammalian TOP2s seem to be specialized evolutionary progeny of an ancestral Top2 like the Top2 of yeast and *Drosophila* [92,93,191]. That Top2, in turn, is an evolutionary descendant of other Type IIA enzymes found in bacteria (e.g., DNA Gyrase, Topoisomerase IV) [192,193]. Mammalian TOP3s likely descend from the Type IA enzymes of yeast and bacteria (Top3 and TopA/TopB, respectively) [194,195]. Indeed, there are several more unique topoisomerases as well, such as archaean Reverse Gyrase which introduces positive supercoils to increase the melting temperature of DNA exposed to extreme temperatures in that system [196,197]. Archaea also have Topoisomerase V, which is classified as a Type IC enzyme, which functions mechanistically like Type IB enzymes (e.g., TOP1) but is structurally unique to Type IA/B enzymes [198,199]. Viruses also often carry topoisomerases, such as Vaccinia Topo, a Type IB enzyme, and T4 Topoisomerase, a Type IIA [200,201]. In short, the field of topoisomerase research is replete with fascinating foci of inquiry.

Within the context of topoisomerase–transcription dynamics, there are many unanswered questions, as discussed in detail in the body of this review. For instance, to what extent does supercoiling propagate from sites of RNAP activity versus being constrained and resolved in the vicinity of RNAPII? Similarly, do chromosome architectural features, such as TADs and chromosome compartments, or the nuclear matrix anchors, in fact, act as boundary elements creating topologically closed domains for supercoiling? Addressing these questions will be important for understanding the potential impact of dynamic supercoiling on chromosome structure and gene regulation. Additionally, innovations that allow for the mapping of positive supercoils and alternative DNA structures should also provide new insights into this issue. Finally, while Figure 3 highlights many of the open questions about transcriptional influence by DSBs, mainly a characteristic of TOP2B, the nuances of gene regulatory functions of TOP1 and TOP3B are less well characterized in mammalian systems and present an essential target for future inquiry.

## Figures and Tables

**Table 1:** Topoisomerase mouse phenotypes.

Topoisomerase	Mouse KO Phenotype
**TOP1**	Embryonic Lethal (~4–16 Cell Stage) [66]
**TOP1MT**	Live, Liver Dysregulation [67,68,69]
**TOP2A**	Embryonic Lethal (~4–8 Cell Stage) [70,71]
**TOP2B**	Perinatal Lethal (Lung, Neuronal Defects) [72]
**TOP3A**	Embryonic Lethal (<E7.5) [73,74]
**TOP3B**	Live, Autoimmunity, Reduced Lifespan [75]

**Table 2:** Topoisomerase inhibitors and poisons.

Drug/Tool	Topo	Type	Step	Mechanism
Camptothecin	TOP1	Poison	Post-cut	Stabilizes TOP1ccs [131]
Irinotecan	TOP1	Poison	Post-cut	Stabilizes TOP1ccs [132]
Topotecan	TOP1	Poison	Post-cut	Blocks Religation [131]
Belotecan	TOP1	Poison	Post-cut	Stabilizes TOP1ccs [131]
Trastuzumab Deruxtecan	TOP1	Poison	Post-cut	Accumulates TOP1ccs [133]
Sacituzumab Govetican	TOP1	Poison	Post-cut	Accumulates TOP1ccs [134]
Indenoisoquinolines	TOP1	Poison	Post-cut	Stabilizes TOP1ccs [135]
Indolocarbazoles	TOP1	Poison	Post-cut	Stabilizes TOP1ccs [12]
DIA-001	TOP1	Poison	Post-cut	Promotes TOP1-DNA Adducts [136]
TOP1^flox^ Mouse Line	TOP1	Conditional Knockout	Transcription	Floxed Allele [88]
TOP1:AID	TOP1	Degron	Pre-binding	Auxin-induced degradation [137]
Quercetin	TOP1/TOP2A/B	Inhibitor/ Poison	Pre-binding/Post-cut	Unknown [138]
Genistein	TOP1/TOP2A/B	Inhibitor/ Poison	Pre-binding/Post-cut	Unknown [138]
Etoposide	TOP2A/B	Poison	Post-cut	Blocks Religation [139]
Teniposide	TOP2A/B	Poison	Post-cut	Blocks Religation [139]
Doxorubicin	TOP2A/B	Poison	Post-cut	Stabilizes TOP2ccs [140]
Daunorubicin	TOP2A/B	Poison	Post-cut	Stabilizes TOP2ccs [141]
Epirubicin	TOP2A/B	Poison	Post-cut	Stabilizes TOP2ccs [142]
Idarubicin	TOP2A/B	Poison	Post-cut	Stabilizes TOP2ccs [140]
Mitoxantrone	TOP2A/B	Poison	Post-cut	Stabilizes TOP2ccs [12]
Ellipticine	TOP2A/B	Poison	Post-cut	Stabilizes TOP2ccs [12]
XK469	TOP2B	Poison	Post-cut	Stabilizes TOP2ccs [143]
Aclarubicin	TOP2A/B	Inhibitor	Pre-binding	Inhibits Decatenation [144]
ICRF-187	TOP2A/B	Inhibitor	Post-bind/Pre-cut	Locks ATPase Clamp [145]
ICRF-193	TOP2A/B	Inhibitor	Post-bind/Pre-cut	Locks ATPase Clamp [146]
TOP2B^flox^ Mouse Line	TOP2B	Conditional Knockout	Transcription	Floxed Allele [147]
TOP2A:AID	TOP2A	Degron	Pre-binding	Auxin-induced degradation [148]
TOP2B:AID	TOP2B	Degron	Pre-binding	Auxin-induced degradation [148]
Bisacridine	TOP3B	Poison	Post-cut	Stabilizes TOP3Bccs [149]
Thiacyanine	TOP3B	Poison	Post-cut	Stabilizes TOP3Bccs [149]
Bemcentinib	TOP3B	Inhibitor	Pre-binding	Inhibits Relaxation [150]
TOP3B^KO^ Mouse Line	TOP3B	Knockout	Transcription	Gene Truncation [123]

## Data Availability

No new data were created or analyzed in this study. Data sharing is not applicable to this article.
